# Inferring the Population Expansions in Peopling of Japan

**DOI:** 10.1371/journal.pone.0021509

**Published:** 2011-06-29

**Authors:** Min-Sheng Peng, Ya-Ping Zhang

**Affiliations:** 1 State Key Laboratory of Genetic Resources and Evolution, Kunming Institute of Zoology, Chinese Academy of Sciences, Kunming, Yunnan, People's Republic of China; 2 KIZ/CUHK Joint Laboratory of Bioresources and Molecular Research in Common Diseases, Kunming, Yunnan, People's Republic of China; 3 Laboratory for Conservation and Utilization of Bio-resources, Yunnan University, Kunming, Yunnan, People's Republic of China; 4 Graduate School of the Chinese Academy of Sciences, Beijing, People's Republic of China; Institut Pasteur, France

## Abstract

**Background:**

Extensive studies in different fields have been performed to reconstruct the prehistory of populations in the Japanese archipelago. Estimates the ancestral population dynamics based on Japanese molecular sequences can extend our understanding about the colonization of Japan and the ethnogenesis of modern Japanese.

**Methodology/Principal Findings:**

We applied Bayesian skyline plot (BSP) with a dataset based on 952 Japanese mitochondrial DNA (mtDNA) genomes to depict the female effective population size (N_ef_) through time for the total Japanese and each of the major mtDNA haplogroups in Japanese. Our results revealed a rapid N_ef_ growth since ∼5 thousand years ago had left ∼72% Japanese mtDNA lineages with a salient signature. The BSP for the major mtDNA haplogroups indicated some different demographic history.

**Conclusions/Significance:**

The results suggested that the rapid population expansion acted as a major force in shaping current maternal pool of Japanese. It supported a model for population dynamics in Japan in which the prehistoric population growth initiated in the Middle Jomon Period experienced a smooth and swift transition from Jomon to Yayoi, and then continued through the Yayoi Period. The confounding demographic backgrounds of different mtDNA haplogroups could also have some implications for some related studies in future.

## Introduction

Peopling of Japan, as well as the formation of modern Japanese, is one of the hottest topics in anthropology, archaeology, linguistics, and genetics in East Asia [1], [2], [3]. Towards the most prevailing “dual structure model”, two major migration events brought different immigrants from the Asian continent to the Japanese archipelago, giving rise to Jomon and Yayoi Cultures, respectively [3]. And modern Japanese were suggested as the result of an admixture between the Jomon and Yayoi populations [3], [4]. This model has been also supported by some recent genetic data, such as mitochondrial DNA (mtDNA) [5], Y chromosome [6], [7], [8], and even ancient DNA [9]. However, the dichotomies between Jomon and Yayoi Cultures had been suggested to be questionable recently [10], implying the process of peopling of Japan would be much more complicated. Investigating the population dynamics during the prehistoric period in Japan, especially around the transition from Jomon to Yayoi Period, would help to further understand the ethnogenesis of modern Japanese (e.g. [11]). Unfortunately, the related archaeological studies are still relative poor and the nature of the Jomon – Yayoi transition remains a matter of continuing scientific debate [12].

The genetic approaches provide a powerful means to investigate the ancient population dynamics of modern human [13], [14], [15], [16]. In recent years, the Bayesian skyline plot (BSP) which based on Bayesian coalescent inference together with a Markov chain Monte Carlo (MCMC; [17], [18]) sampling algorithm has been introduced to estimate past effective population dynamics through time from a set of DNA sequences [19]. Besides not depending on a prespecified parametric model of demographic history, the BSP takes into account both the error inherent in phylogenetic reconstruction and the stochastic error intrinsic to the coalescent process, and allows more complex demographic trends to be identified [19], [20]. These characters make the BSP as a useful tool to reconstruct prehistoric demographics of modern human, especially based on mtDNA genome variation which was suggested to be a good predictor of population size in humans [21]. And these studies had extended our understandings about the prehistoric population dynamics in Africa [22], [23], Asia [21], [24], [25], and North America [26], [27], [28], [29].

During the past decade, data of Japanese mtDNA genomes have been accumulated quickly (e.g. [30]). As increasing the sample size could improve the accuracy in the coalescent analyses of population genetic data [26], [31], the massive available mtDNA data may provide us an opportunity to reconstruct the prehistoric demographics of Japanese in more detail. To this end, we incorporated 952 Japanese mtDNA coding region sequences, and then preformed the BSP to estimate the changing of female effective population size (N_ef_) through time.

## Results

**Figure 1:**
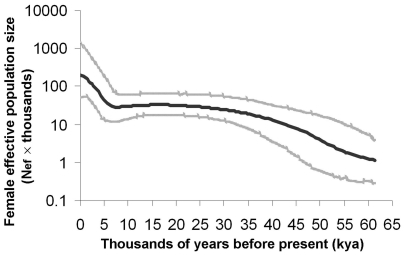
BSP of female effective population size (N_ef_) through time for total Japanese mtDNA lineages. The bold black line represents the median population size estimate from Bayesian posterior distribution. The grey lines delimit the 95% highest posterior density (HPD) boundaries, accounting for uncertainty in the reconstructed phylogeny and substitution model parameters. N_ef_ is plotted on a log scale and assumes a generation time of 20 years [66]. All the lines were drawn within the lower 95% HPD boundary of the maximum time to the coalescent age.

Figure 1 (also Figure S1) shows the BSP of N_ef_ through time for the total Japanese mtDNA lineages (see Material and Methods for detail). The earliest population growth took place ∼55 kya (thousand years ago), which was largely in agreement with the demographic scenario previously observed in the BSP of populations in North and Central Asia [21]. The profiles of mtDNAs in Japanese showed similar structure with the other populations in Asian continent [5], [30], [32]. Especially, many mtDNA lineages in Japanese could be traced back to the origins in North and/or Central Asia [30]. As a result, the initial population growth happening ∼55 kya which was much older than the appearance of modern human in Japanese archipelago [33], was most likely to be attributed to the common background of ancient demographics in North and Central Asia. Later, around the Last Glacial Maximum (LGM; ∼26.5–19 kya) [34], the population growth began to slow, which might reflect the contemporary adverse climatic conditions. On a more recent timescale, a rapid population expansion since ∼5 kya, which was absent in the backgrounds in North and Central Asia [21], was revealed in the BSP of Japanese.

**Figure 2:**
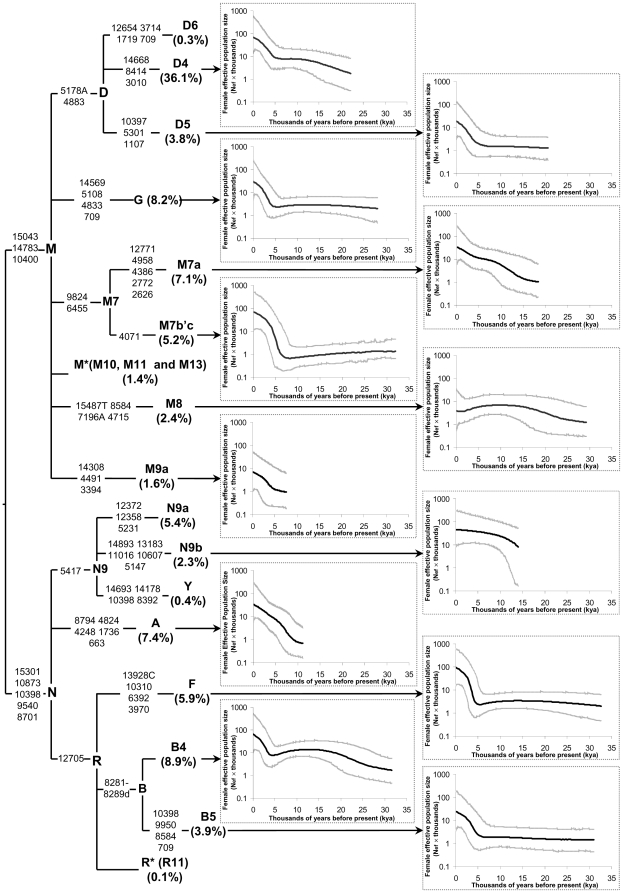
Phylogeny and BSPs of female effective population size (N_ef_) through time for the major Japanese mtDNA haplogroups. The percentage of each haplogroup is indicated in the brackets. For detailed information, please refer the legend of Figure 1.

To infer more details about the demographics of the ancestors of Japanese, we dissected the BSP analyses at the level of mtDNA haplogroups [22]. All 952 mtDNAs could be assigned into the known major haplogroups in the context of East Asians [35] and the phylogeny was shown in Figure 2. Haplogroups D6, M10, M11, M13, R11, and Y, which together made up ∼2.2% of the Japanese mtDNAs, were disregarded in the BSP constructions because of the limited sizes of sequences. As a result, around 97.8% of Japanese mtDNAs which could be assigned into haplogroups D4, D5, G, M7a, M7b'c, M8, M9a, A, N9a, N9b, F, B4 and B5 were adopted in the BSP analyses.

The salient scenario identified in the BSP analyses for the major mtDNA haplogroups was a rapid increasing N_ef_ since around 5 kya (Figure 2), which was coincident with the pattern observed in total Japanese (Figure 1). This pattern was seen in haplogroups D4, D5, G, M7b'c, F, B4 and B5 which together accounted for ∼72% Japanese mtDNAs. Before this recent pronounced population growth, the more ancient demographics of these haplogroups presented some different patterns. For instance, a long nearly stasis of population growth was shown in haplogroups D5 and B5; whereas a small population size drop was detected in haplogroups M7b'c and B4. For the rest ∼20% mtDNA lineages, the separate BSPs for haplogroups M7a, A, M9a, M8, and N9b were different from the above. Examination of the plots in haplogroups M7a, A, and M9a indicated gradual, roughly exponential growth occurring around the end of the LGM, the beginning, and the middle of the Holocene, respectively. For haplogroup M8, the slight fluctuation of N_ef_ through time but without recent expansion was observed. The plots in haplogroup N9b showed gradual and slow growth of N_ef_ within the past ∼15 kya. Unfortunately, the BSP for haplogroup N9a (∼5.4%) presented a very weird pattern: very low N_ef_ value and very short coalescent time estimation (data were not shown). This result might suggest that the demographic history of haplogroup N9a could not be constructed under the current model of the BSP, and we excluded this haplogroup in further discussion.

## Discussion

Our results indicated that the recent rapid population growth since ∼5 kya as a major force in shaping the current maternal gene pool of Japanese. This demographic event had left a pronounced genetic imprint to distinguish Japanese from the common background in North and Central Asia. Further analyses revealed that this imprint was presented in ∼72% Japanese mtDNAs from seven haplogroups (i.e. D4, D5, G, M7b'c, F, B4 and B5) with different backgrounds (including the estimated coalescent ages, the proposed origins and geographic distributions) [30], suggesting it was unlikely to be provoked by multiple simultaneous selective sweeps on different haplogroups.

Although our results might only reflect the maternal history of Japanese, they were supported by some parallel evidence from archaeology, and shed some light into the issue about the transition from Jomon to Yayoi. The timing of the recent pronounced population expansion was overlapping with the Middle Jomon Period (ca. 3700–2600 B.C.) [36] when some archaeological researches revealed that the Jomon people extended the exploitation of plant food (e.g. acorns and horse chestnuts) and marine resources [36], even carried out some management of environments and species [37], [38]. As a result, the organizational complexity in subsistence and settlement reached its high point (e.g. Sannai Maruyama site) [36], [39], and the population size was suggested to undergo an outstanding expansion [11], [12]. Interestingly, our results showed that the population growth since ∼5 kya was lasting to the present, suggesting a smooth and swift transition from Jomon to Yayoi and then the subsequent population expansion in the Yayoi Period [40]. Although there're some arguments [39], plants domestication (e.g. barnyard grass and bottle gourd) was suggested to have occurred in Japan at least by the Middle Jomon Period [37], [41]. So the advanced knowledge and technology introduced by some Yayoi immigrants, such as rice paddy agriculture, could be quickly appropriated by the local Jomon populations who might already have some experiences in agriculture production [40].

Despite these promising results, whether the genetic signal would be affected by other demographic factors, such as migration and admixture, is still elusive. Indeed, as the prehistoric intensive agriculture had been set up ∼5 kya (e.g. the development of irrigated rice systems [42], [43]), there were major increases in the quantity of sites and average site size, both attesting to demographic growth, in the Late Yangshao and even more so in the Longshan Period in central China [36], [44], [45]. In Korea, although it was suggested that broomcorn and foxtail millets were established no later than 3400 B.C. (the Middle Chulmun) [46], the present picture of Chulmun (6000–1500 B.C.), including population densities and social complexity, is far from clear [36] [39]. Thus, it's possible that the pronounced population growth ∼5 kya might not take place in Japan but rather the neighboring Asian continent; and then the related signal of expansion could be brought to Japan due to the later population migration from the continent. To infer the migration as well as admixture, large mitochondrial genome data from the potential “source populations” in the continent (e.g. China and Korea) are required. Unfortunately, these data based on whole population samplings are still absent.

It also should be noted that, other factors in BSP can not be ignored simply. One is that the timescale estimation in the BSP depends on the mutation rate used (e.g. [27], [47]). In this study, we adopted the mutation rate as 1.691×10^−8^ substitutions per site per year, because this rate seemed moderate to date the prehistoric expansion events for global human populations as compared with phylogeographic analyses [21]. When using the faster rate (e.g. [48]), the time estimates of the rapid population expansion in Japan would be more recent, which could be attributed to the dominant role of Yayoi expansion through demic and/or cultural diffusion. Meanwhile, the limitations of the temporal resolution of the current BSP method and mtDNA data might hamper to distinguish population dynamics in a short time [21]. Moreover, the transitions from Jomon to Yayoi showed as different patterns between western (e.g. northern Kyushu) and eastern Japan (e.g. Tohoku) [3], [37], which might be reflected in some genetic differentiation among different regions [49]. To resolve these issues, the improved method with more explicit model (e.g. the Extended Bayesian Skyline Plot [50]), together with multiple genetic data (e.g. genome-wide SNPs [49]) covering wide regions and multiple approximate internal calibration points (e.g. [23], [48], [51]), are needed in the future.

In addition to indicating the pronounced recent population expansion, we identified some different prehistoric demographic history of different mtDNA haplogroups. For example, as a characteristic mtDNA lineage mainly concentrating in Japanese [30], [52], haplogroup M7a indicated a progressive exponential population growth shortly after the end of the LGM, which could be attributed to some successful adaptive subsistence strategies well suited to the changing post-LGM environment [40]. Taken together (as well as haplogroup N9a), our results revealed the heterogeneous demographic background of the mtDNA pool in current Japanese. And the demographic effects on the related studies (e.g. the work about association between certain mtDNA haplogroup and disease) should be examined in the future.

## Materials and Methods

### Sequence data

We retrieved the published Japanese (neither Ainu nor Ryukyuan) mtDNA genomes from GenBank, and collected 954 mtDNA genomes in total (including five sequences with coding region only; Table 1) [30], [53], [54], [55], [56], [57], [58], [59]. The data were suggested to be from different individuals but not focused on particular haplogroups. Through quality control as described previously [60], [61], eleven sequences (GenBank Accession Numbers: AP008259, AP008269, AP008278, AP008306, AP008552, AP008776, AP008777, AP008798, AP008799, AP008801, and AP008803) were confirmed as artificial recombinants [60]; two sequences (AP008253 and AP008308) were suspected as potential artificial recombinants [60]; and two (AP008336 and AP008866) were suggested to have editing errors [61]. AP008253 and AP008308 were excluded in any further analyses. The other 13 sequences were corrected [60], [61] and could be retrieved from PhyloTree (http://www.phylotree.org/; [35]). So we replaced the original sequences with the corrected ones which were noted as “CORRECTED”. Through the manually diagnostic motif search, we assigned each of the 952 mtDNAs into specific haplogroup (Table S1). And we employed MitoTool (http://www.mitotool.org/; [62]) to check the haplogroup status of the sequences, which based on mutation motif(s) matches with PhyloTree. Referring the revised Cambridge Reference Sequence (J01415.2; [63]), thirteen protein coding genes were extracted, concatenated, and then aligned in ClustalX [64], with the *ND6* gene readjusted to present the same reading direction as the other genes.

**Table 1:** General information for Japanese mtDNAs used in this work.

References	Size	Accession Numbers	Sources
[30]	672	AP008249-AP008920	from Japanese individuals sampled in Tokyo and the Nagoya area
[53]	2	AF346989-AF346990	from Japanese individuals
[54]	57	AP009419-AP009475	from individuals in Japan without ethnic information
[55]	5	DQ112864-DQ112868	from individuals in Japan without ethnic information
[56]	112	AP010661-AP010772	from 112 semi-supercentenarians across Japan
[57]	2	EU597488, EU597571	from Japanese individuals
[58]	90	AP010970-AP011059	from 93 Japanese patients with schizophrenia
[59]	14	AP010824-AP010837	from 100 healthy unrelated Japanese across Japan
**Total**	954	—	—

### Bayesian skyline plots

For each of the major mtDNA haplogroups in Japanese, BSPs for N_ef_ through time were constructed using BEAST v1.4.8 (http://beast.bio.ed.ac.uk/; [65]) as described before [21], [22]. In detail, we adopted a general time-reversible (GTR) substitution model with site-specific rates for first, second and third codons to infer the ancestral gene trees for each haplogroup. In order to estimate the time scale to the N_ef_ changing, we chose strict molecular clock with the fixed rate as 1.691×10^−8^ substitutions per site per year. Considering the computational load and the convergence of MCMC, we constructed the BSP for a dataset of 100 sequences randomly sampled from the entire 952 mtDNAs to estimate the N_ef_ for total Japanese through time. Similarly, for the most dominant haplogroup – haplogroup D4, we randomly sampled 100 sequences from the 340-sequence dataset to depict the BSP. These computations were repeated with another independent sampling and the results were consistent (Figures 1, 2 and S1, S2). Some rare haplogroups with very few sequences were neglected in the analyses. Each MCMC was run for 40,000,000 generations and sampled every 4,000 generations with the first 40,000 generations discarded as burn-in. The input file for the BSP was prepared by BEAUti within the package of BSEAT. In all runs, the effective sample size values for the parameters of interest were over 500. The results were visualized with Tracer v1.5 (http://tree.bio.ed.ac.uk/software/tracer/).

## Supporting Information

Figure S1 **BSP of female effective population size (N_ef_) through time for total Japanese mtDNA lineages.** This result was generated by additional randomly sampling of 100 sequences.
(TIF)

Figure S2 **BSP of female effective population size (N_ef_) through time for Japanese mtDNA haplogroup D4 lineages.** This result was generated by additional randomly sampling of 100 sequences.
(TIF)

Table S1 List of 952 mtDNA genomes identified in modern Japanese. This file described the haplogroup status and the reference information about 952 Japanese mtDNA genomes.
(XLS)
