# Supplementary figures and images for: Inferring the Population Expansions in Peopling of Japan
Source: PLoS One. 2011 Jun 29;6(6):e21509. doi: 10.1371/journal.pone.0021509 (PMC3126835; doi:10.1371/journal.pone.0021509)

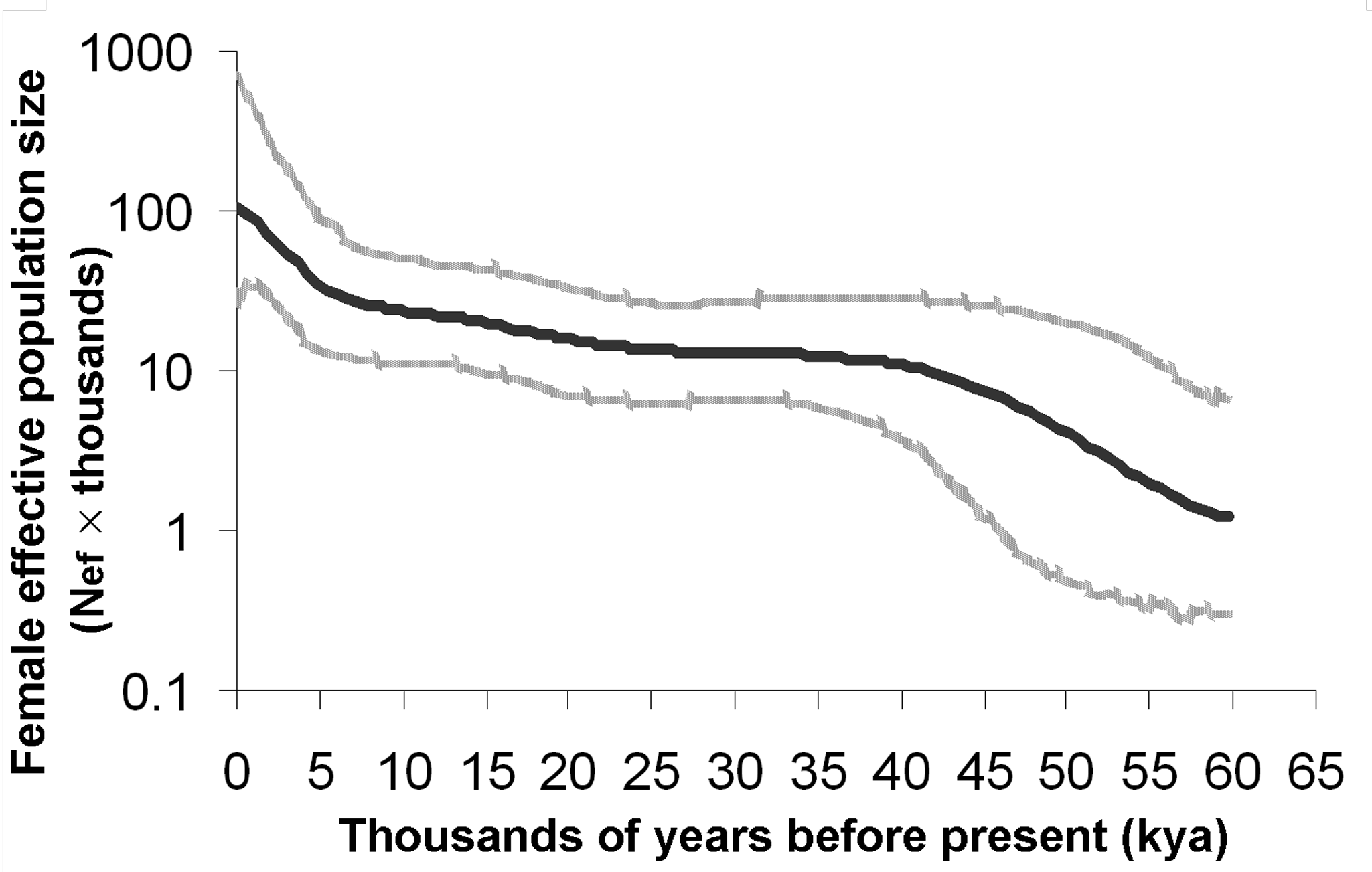

Supplement: Figure S1 — BSP of female effective population size (Nef) through time for total Japanese mtDNA lineages. This result was generated by additional randomly sampling of 100 sequences. (TIF) [file pone.0021509.s001.tif]

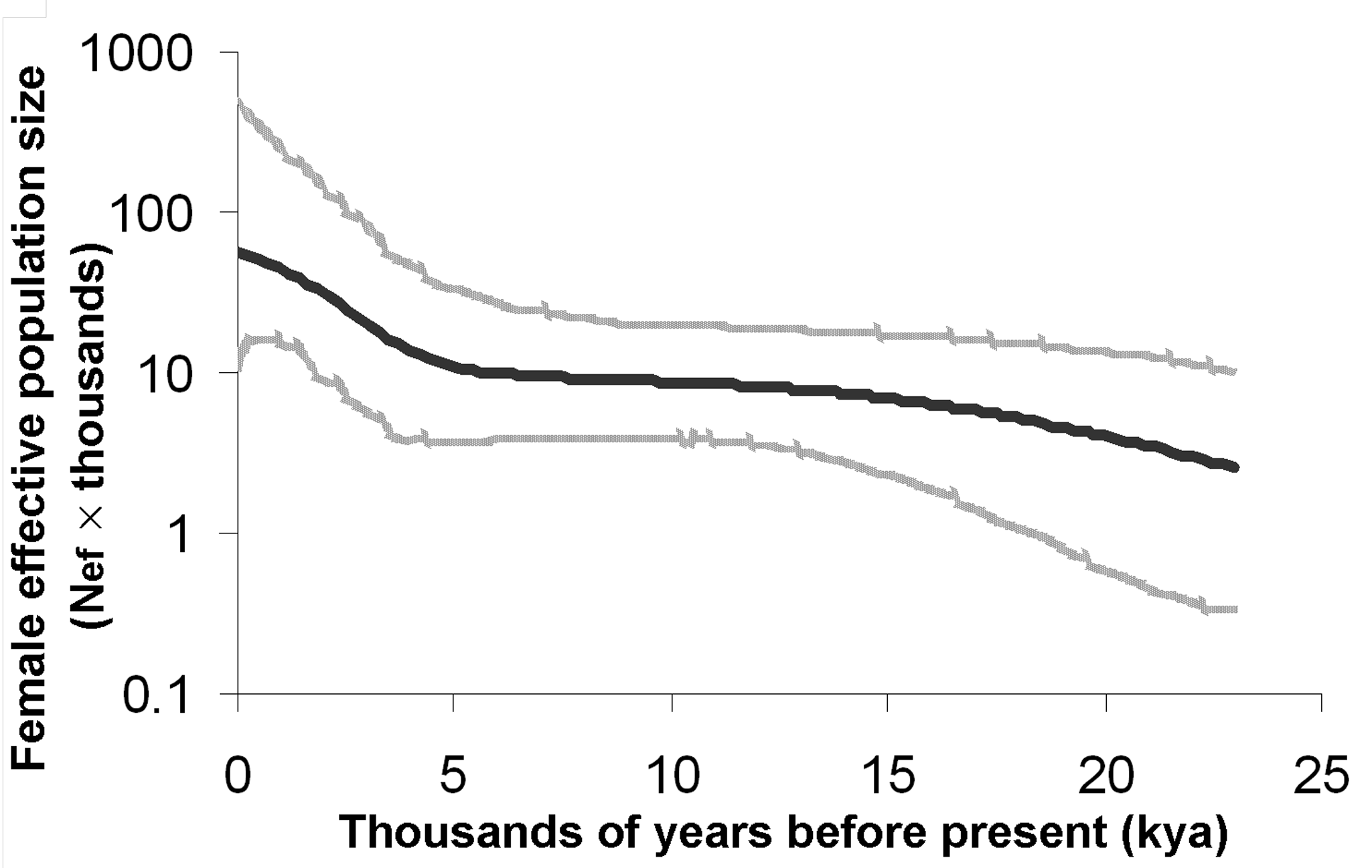

Supplement: Figure S2 — BSP of female effective population size (Nef) through time for Japanese mtDNA haplogroup D4 lineages. This result was generated by additional randomly sampling of 100 sequences. (TIF) [file pone.0021509.s002.tif]
